# Supplementary material for: Necroptosis stimulates interferon-mediated protective anti-tumor immunity
Source: Cell Death Dis. 2024 Jun 10;15(6):403. doi: 10.1038/s41419-024-06801-8 (PMC11164861; doi:10.1038/s41419-024-06801-8)
Supplement: Supplementary file 1 — Supplementary figures and legends [file 41419_2024_6801_MOESM1_ESM.pdf]

Supplemental Figure 1

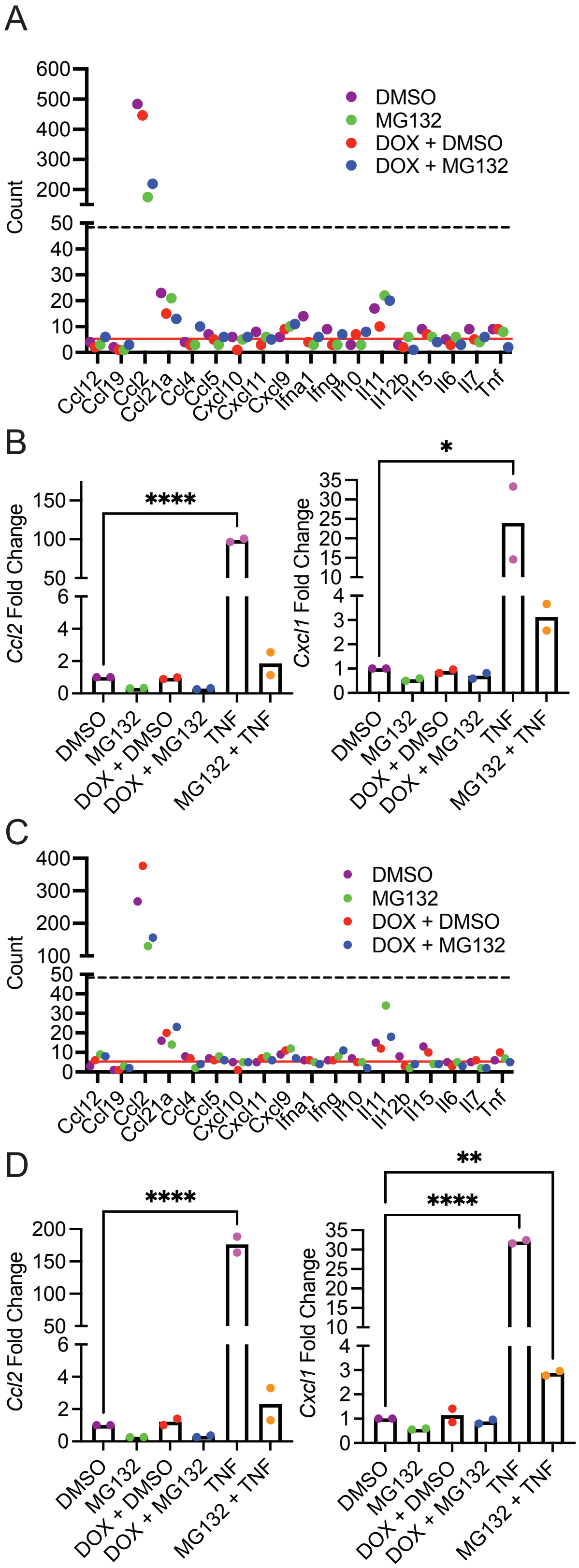

Supplemental Figure 1. Cytokine expression in Casp8- and MLKL-deficient tumor cells. **(A-D)** Casp8-KO and MLKL-KO LLC-OVA cells were treated for 8 hours with DOX (1 μg/mL) followed by treatment with MG132 (4 μM). Three hours after treatment with MG132, RNA was prepped using tumor cell lysates. **(A, C)** Tumor cytokine expression was measured via Nanostring using the mouse tumor 360 signaling panel for **(A)** Casp8-KO cells and **(C)** MLKL-KO cells. The red solid line indicates the mean read count for the negative controls and the dashed black line indicates the mean read count for the lowest positive control. Data is from a single experiment. **(B, D)** Gene expression for *Ccl2* and *Cxcl1* was assessed by qPCR 4.5 hours following treatments as indicated for **(B)** Casp8-KO cells and **(D)** MLKL-KO cells. Each point represents an average of technical replicates from an individual experiment.

Supplemental Figure 2

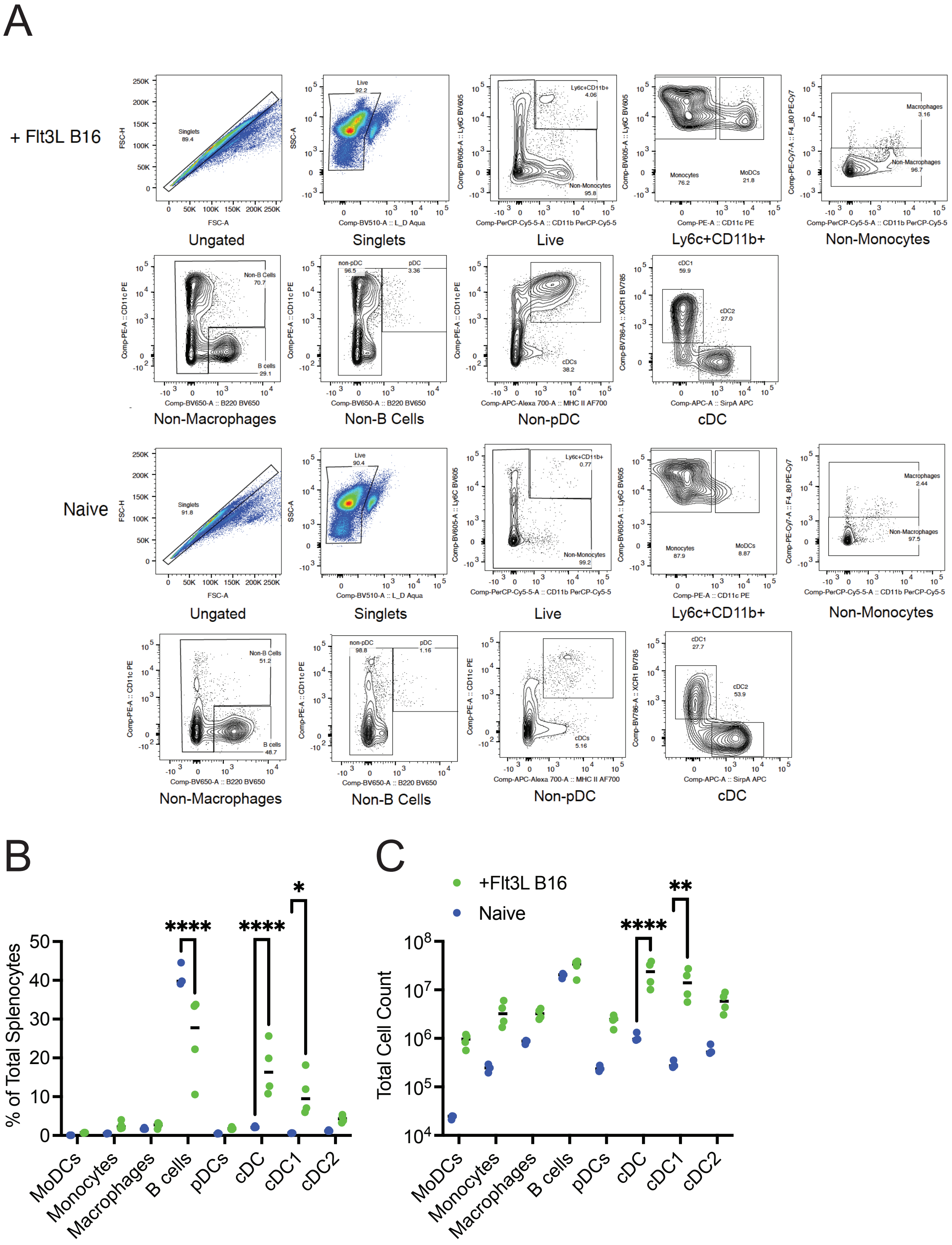

Supplemental Figure 2. Expansion of splenic myeloid populations using *Flt3L*-expressing B16 tumors. (A) Flow gating for splenic myeloid cells for B16-Flt3L tumor-bearing mice and naïve controls. **(B-C)** Quantification of **(B)** % of total splenocytes and **(C)** total numbers of splenic myeloid populations in B16-Flt3L tumor-bearing mice and naïve controls. For **(B, C)**, treatment groups were compared using one-way ANOVA. *P < 0.05, **P < 0.01, ***P < 0.001, and ****P < 0.0001.

Supplemental Figure 3

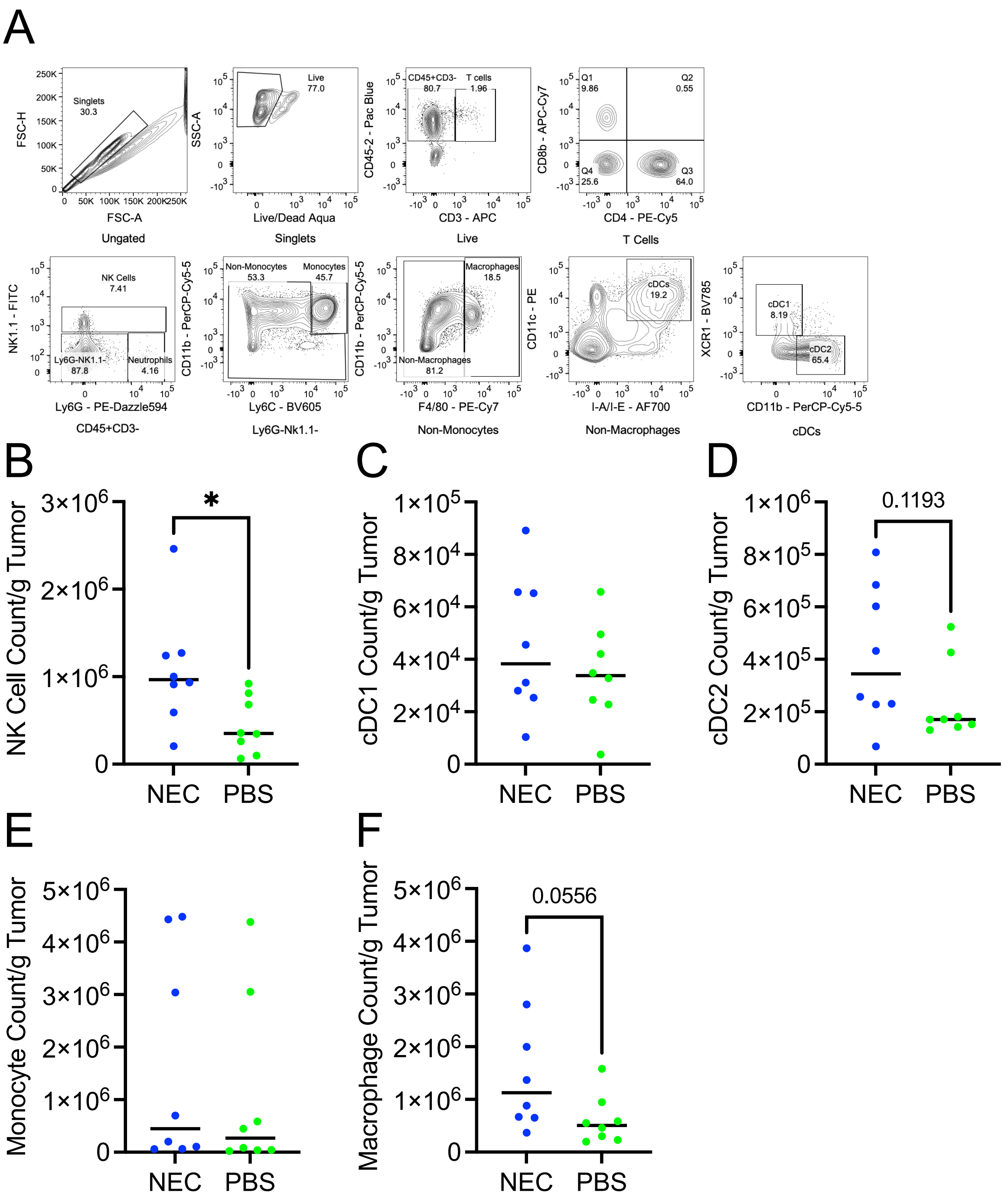

Supplemental Figure 3. Tumor NK cells, but not tumor myeloid cells, are increased in early tumors following NEC immunization. (A) Flow gating for tumor immune cells. Quantification of total count per gram of tumor for **(B)** NK cells, **(C)** cDC1s, **(D)** cDC2s, **(E)** monocytes, and **(F)** macrophages from tumors of NEC or PBS immunized mice at day 5 post tumor challenge. Data is aggregated from two independent experiments (n = 4-5 per treatment group per experiment). For **(B-F)**, treatment groups were compared using unpaired Student's t-test. *P < 0.05, **P < 0.01, ***P < 0.001, and ****P < 0.0001.

Supplemental Figure 4

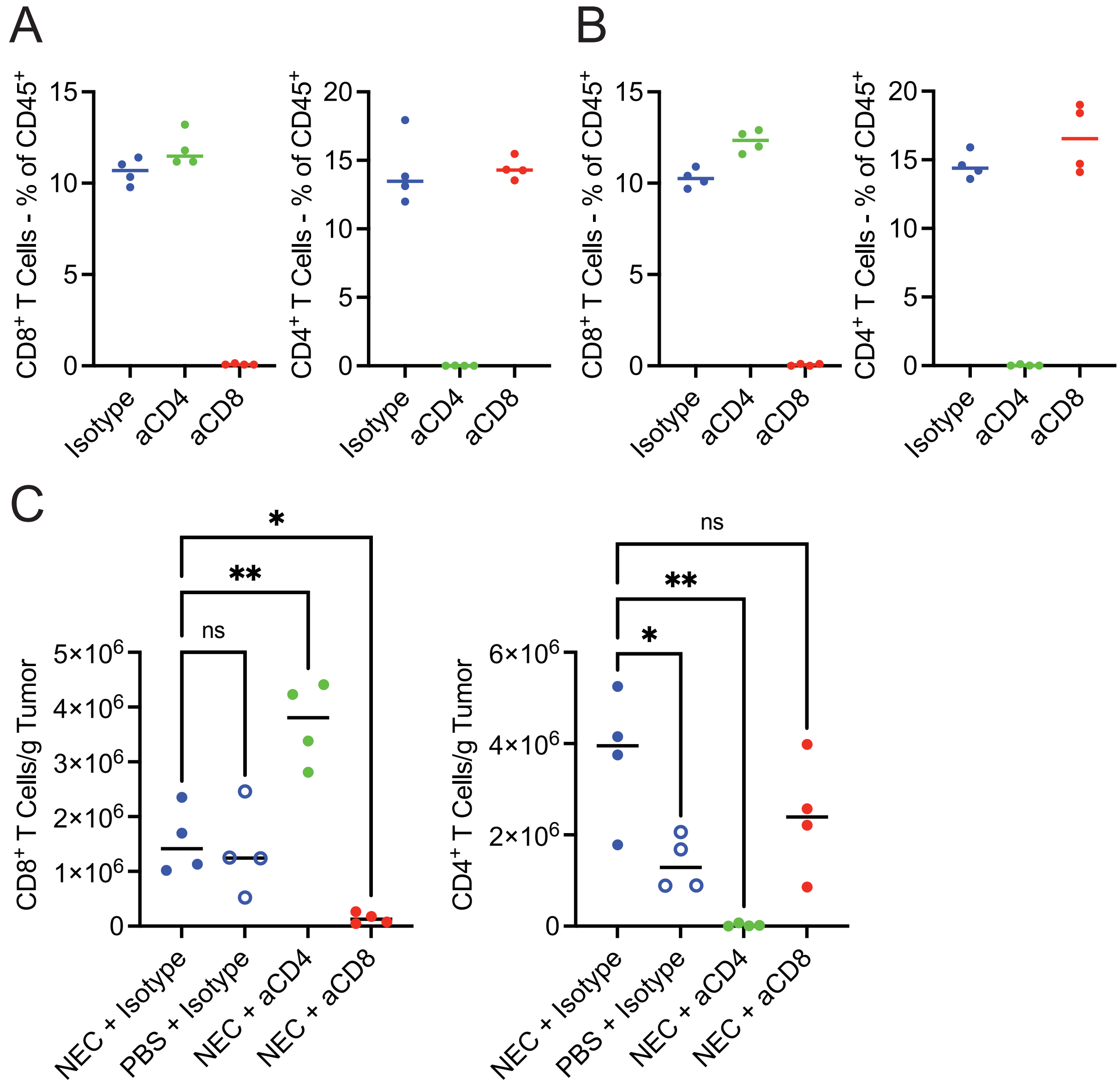

Supplemental Figure 4. Long term T cell depletion following treatment with anti-CD8 or anti-CD4 antibody. Prior to dying cell immunization, mice were treated with anti-CD8a, anti-CD4, or isotype control antibody. **(A-B)** To verify T cell depletion, mice were bled on the day of immunization and tumor challenge. Quantification of $CD8^+$ and $CD4^+$ T cells in the blood as a percentage of $CD45^+$ cells on the **(A)** day of immunization and **(B)** day of tumor challenge. **(C)** T cell infiltrate was assessed in tumors at day 14 post-tumor challenge. Treatment groups were compared using one-way ANOVA. *P < 0.05, **P < 0.01, ***P < 0.001, and ****P < 0.0001. For **(A-C)**, data is representative of two independent experiments (n = 4-5 per treatment group per experiment).

Supplemental Figure 5

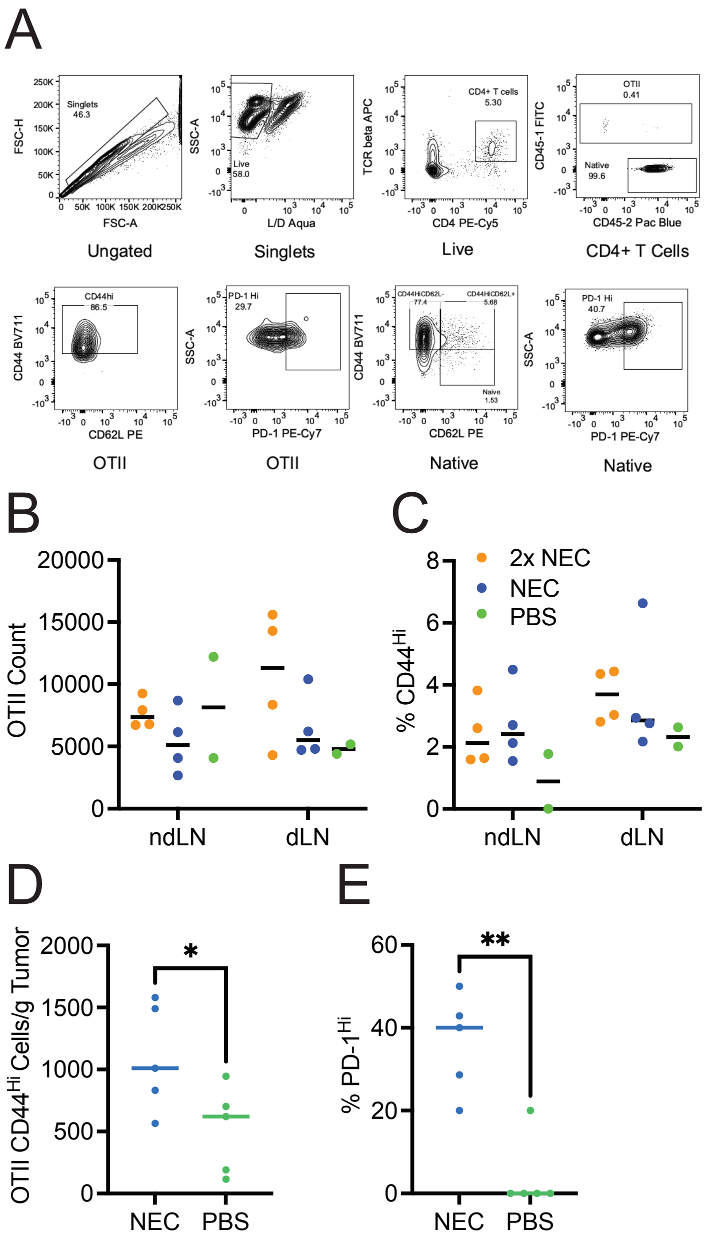

Supplemental Figure 5. Priming tumor antigen-specific T cells following NEC immunization primarily occurs upon tumor challenge and not directly following immunization. (A) Gating strategy for identifying transferred OT-II $CD4^+$ T cells. **(B-C)** OT-II $CD4^+$ T cells were transferred the day prior to NEC immunization. A subset of NEC immunized mice received a second immunization two days later (2x NEC). The immunization site draining lymph node (dLN) and contralateral inguinal lymph node (ndLN) were collected for analysis with flow cytometry. **(B)** Total count and **(C)** % of $CD44^{Hi}$ for OT-II $CD4^+$ T cells found in the lymph nodes as indicated. Data is representative of two independent experiments (n = 2 – 4 per treatment group per experiment). Treatment groups were compared using one-way ANOVA. **(D-E)** Mice were immunized with NEC followed by transfer of OT-II $CD4^+$ T cells 7 days later. Mice were subsequently challenged with live tumor the following day. Tumors were harvested for analysis with flow cytometry. **(D)** Total count of tumor $CD44^{Hi}$ OT-II $CD4^+$ T cells per gram of tumor. **(E)** % of PD-1^{Hi} OT-II cells in tumor. Data is from a single independent experiment (n = 5 per treatment group). For **(D-E)**, treatment groups were compared using unpaired Student's t-test. *P < 0.05, **P < 0.01, ***P < 0.001, and ****P < 0.0001.

Supplemental Figure 6

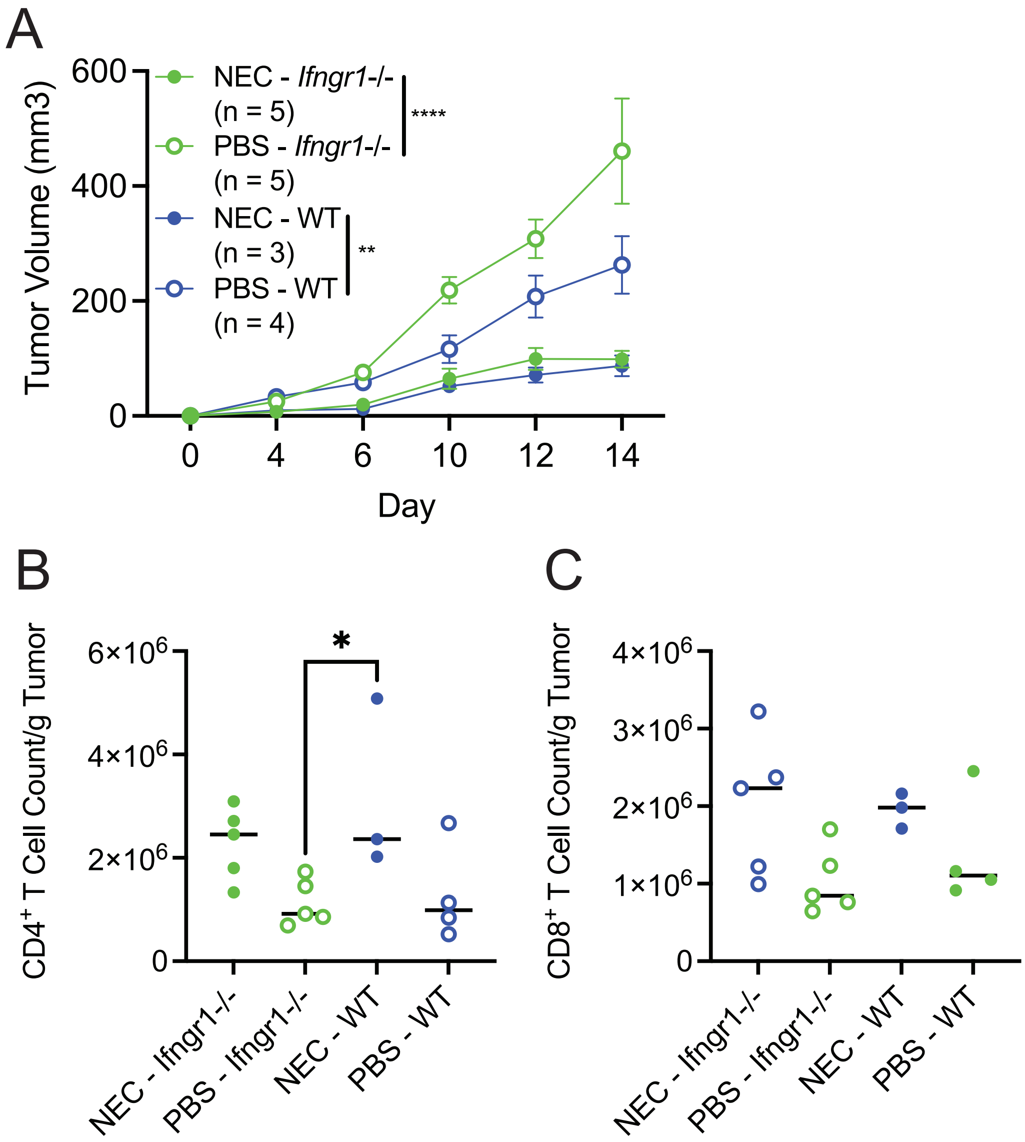

Supplemental Figure 6. Anti-tumor immunity induced by necroptotic dying cells is not lost with host interferon-gamma receptor deficiency. (A-C) Dying cell immunization and subsequent live tumor challenge was performed in WT or *Ifngr1*$^{-/-}$ mice. Data is from a single independent experiment (n = 3-5 per treatment group). **(A)** Tumor volumed was assessed. **(B-C)** T cell infiltrate was assessed in tumors at day 14 post-tumor challenge in WT or *Ifngr1*$^{-/-}$ mice. Total counts of **(B)** CD4^{+} T cells and **(C)** CD4^{+} T cells per gram of tumor. For **(A)**, treatment groups were compared using two-way ANOVA. For **(B-C)**, treatment groups were compared using one-way ANOVA. *P < 0.05, **P < 0.01, ***P < 0.001, and ****P < 0.0001.
