# Supplementary material for: Necroptosis stimulates interferon-mediated protective anti-tumor immunity
Source: Cell Death Dis. 2024 Jun 10;15(6):403. doi: 10.1038/s41419-024-06801-8 (PMC11164861; doi:10.1038/s41419-024-06801-8)

Figure 1a

RIPK3

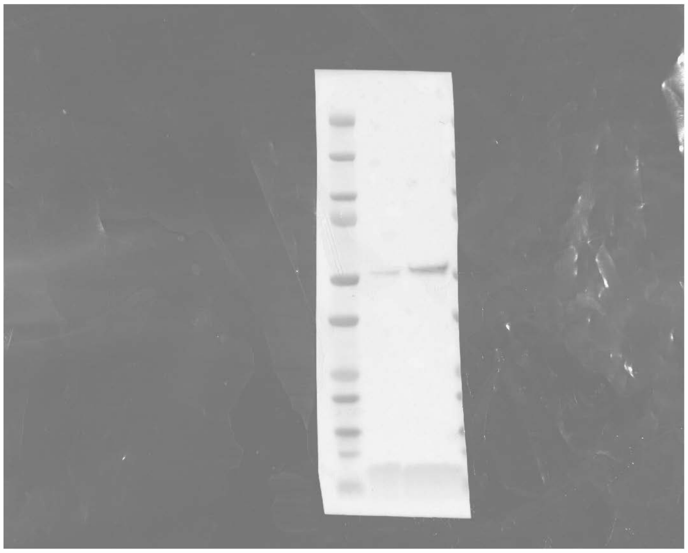

Actin

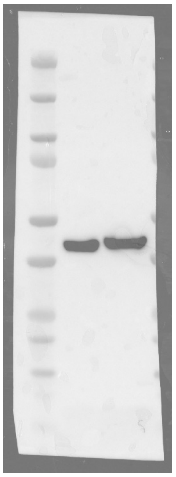

Figure 1c

pMLKL

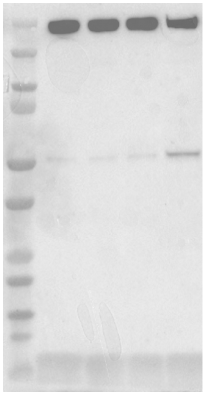

MLKL

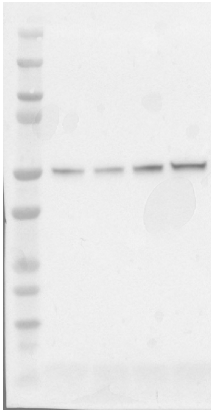

RIPK3

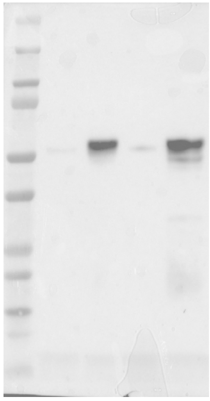

Actin

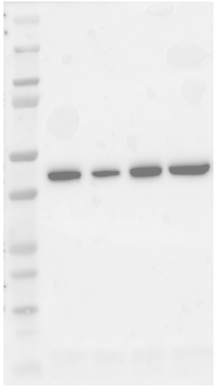

Figure 1e

pMLKL

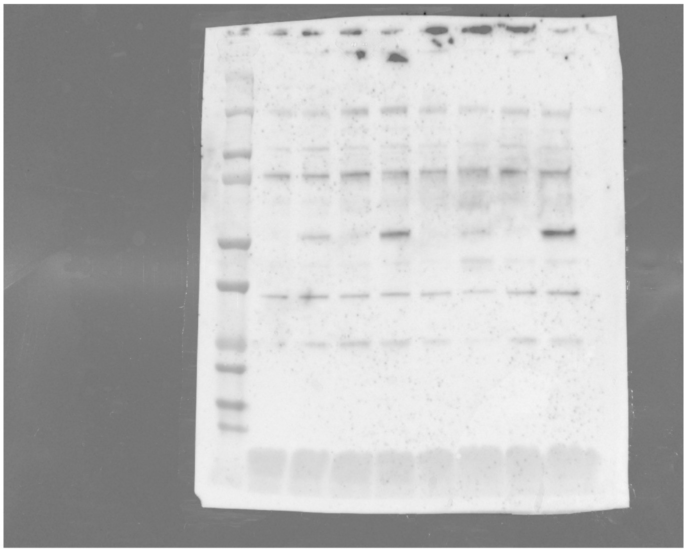

MLKL

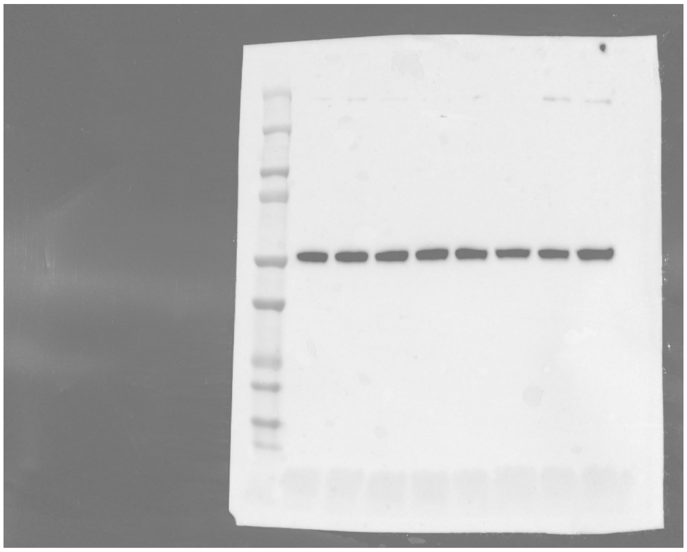

RIPK3

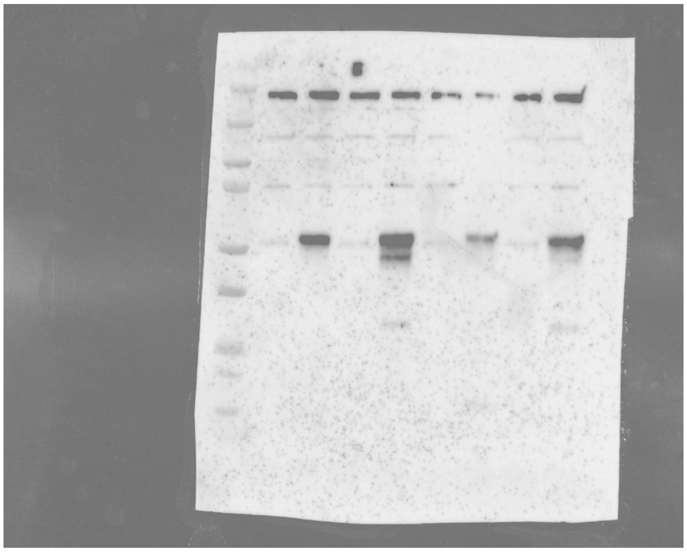

Cleaved Casp3

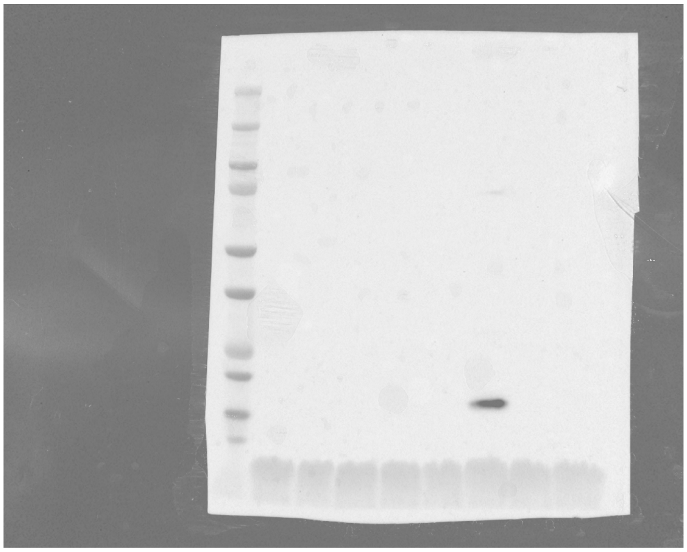

Actin

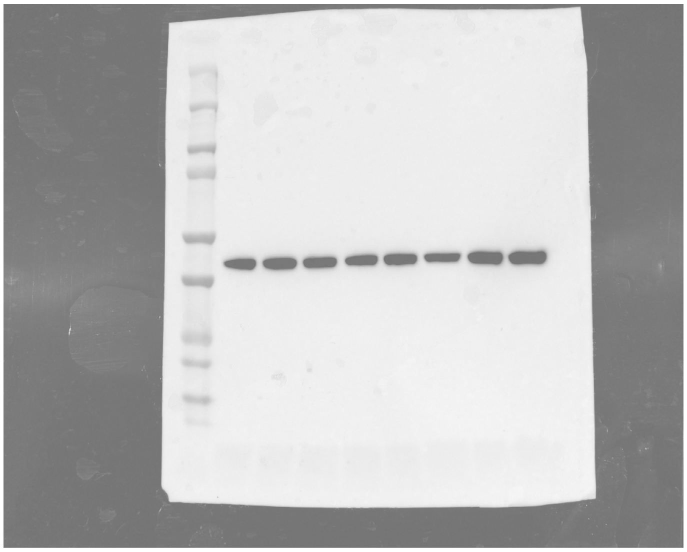

Figure 1f

Poly-K48 Linkage

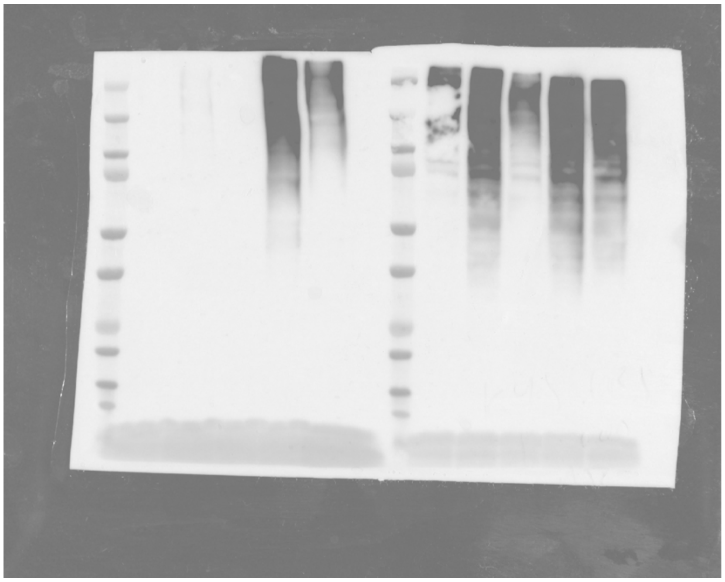

CO-IP on Left, WCL on Right. For each, lane 5 is DOX + MG132 for 2 hours.

FLAG (Clarity)

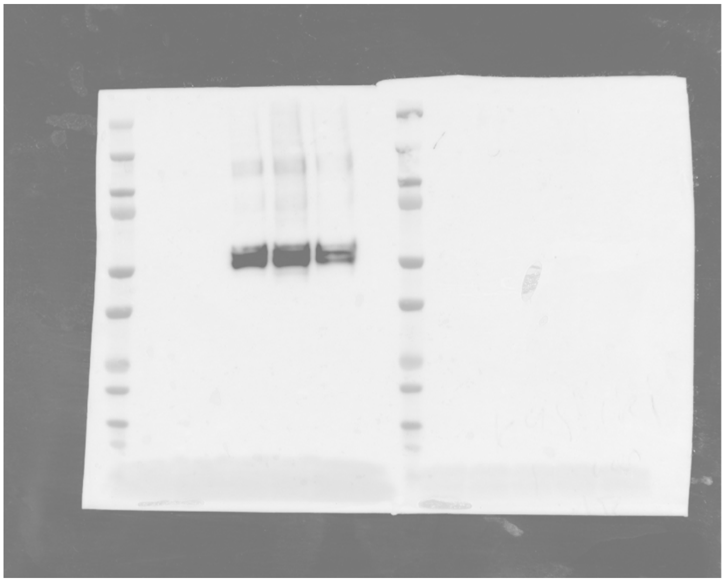

FLAG (Clarity Max)

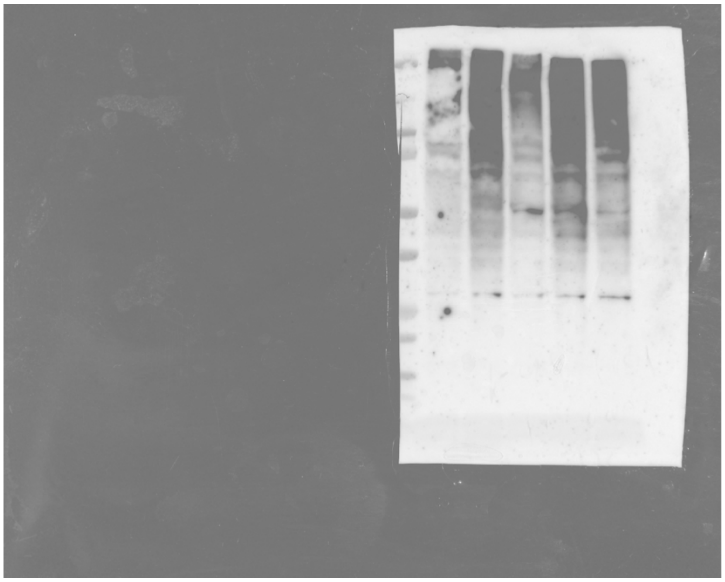

Figure 1h

pMLKL

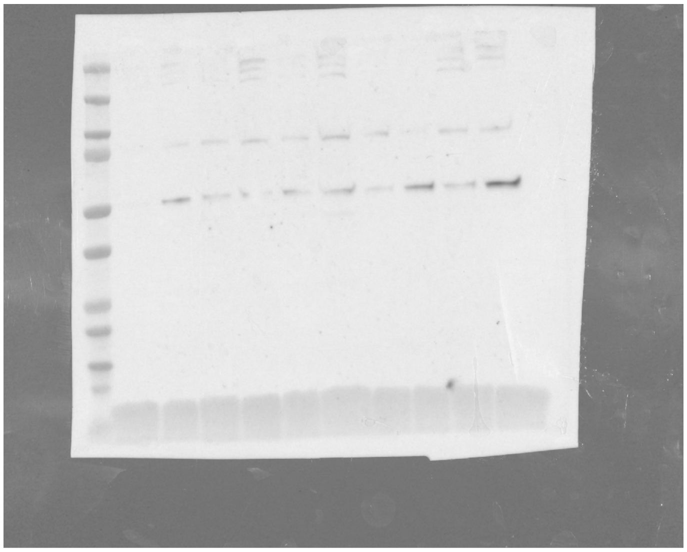

MLKL

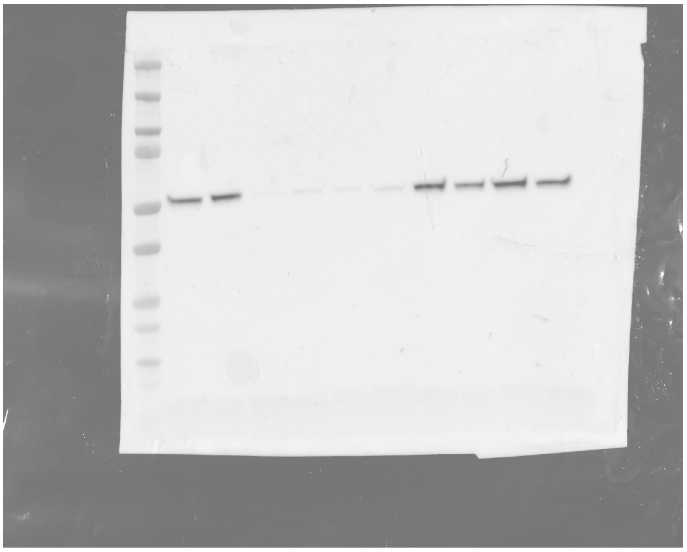

Casp8

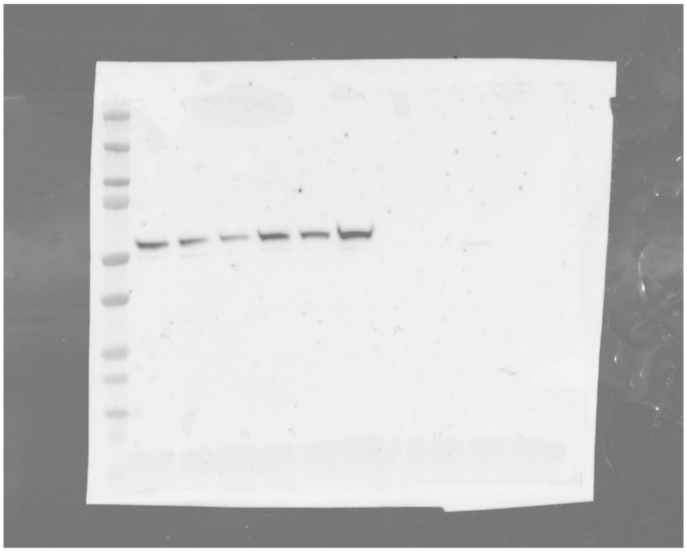

Cleaved Casp3

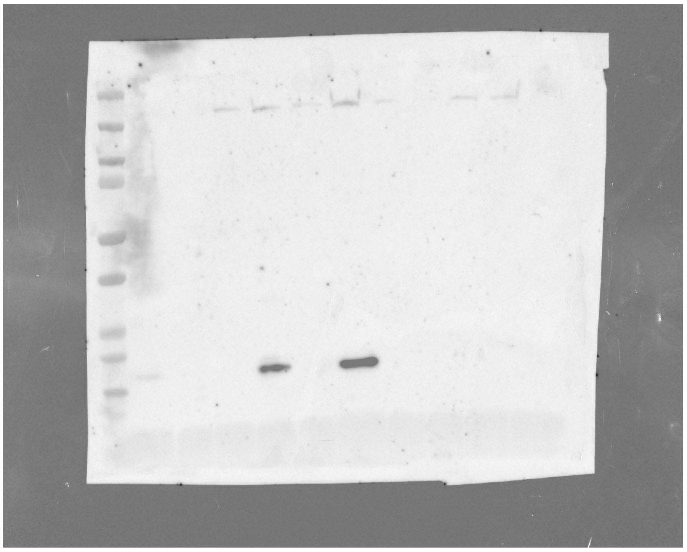

RIPK3

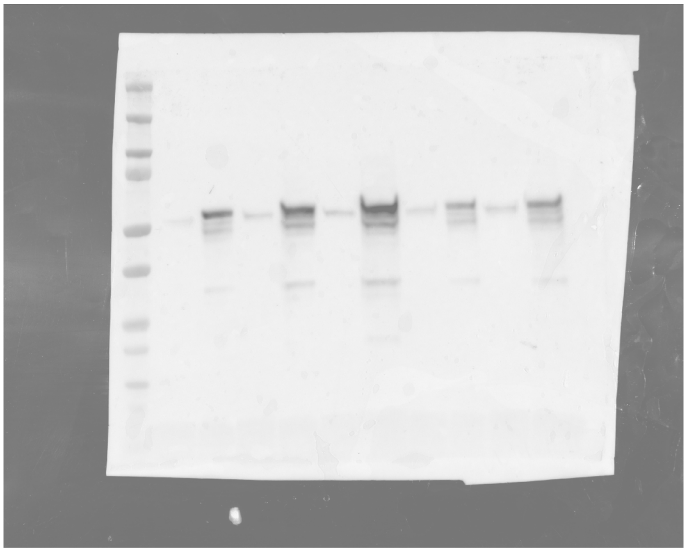

Actin

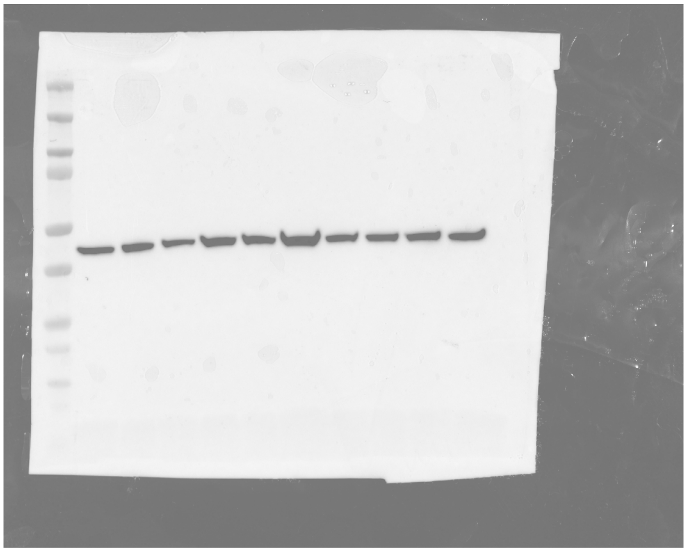

Figure 1j

MLKL

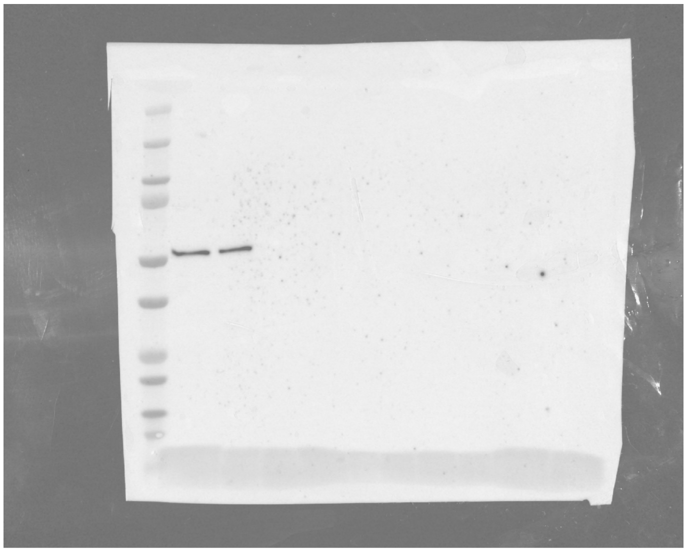

Casp8

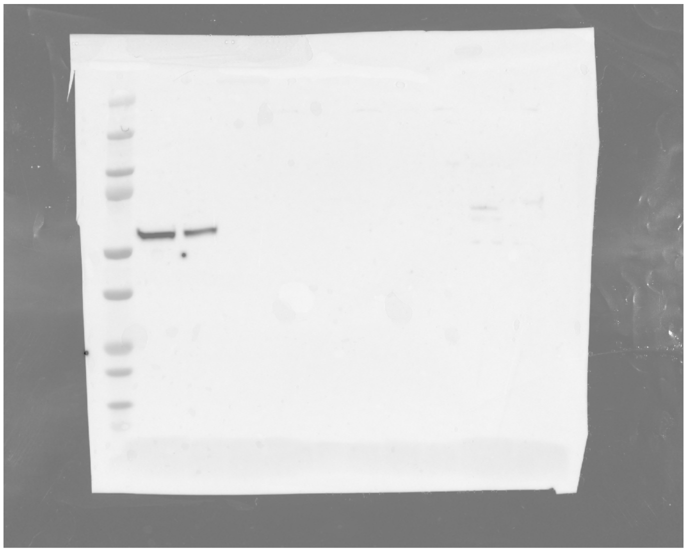

RIPK3

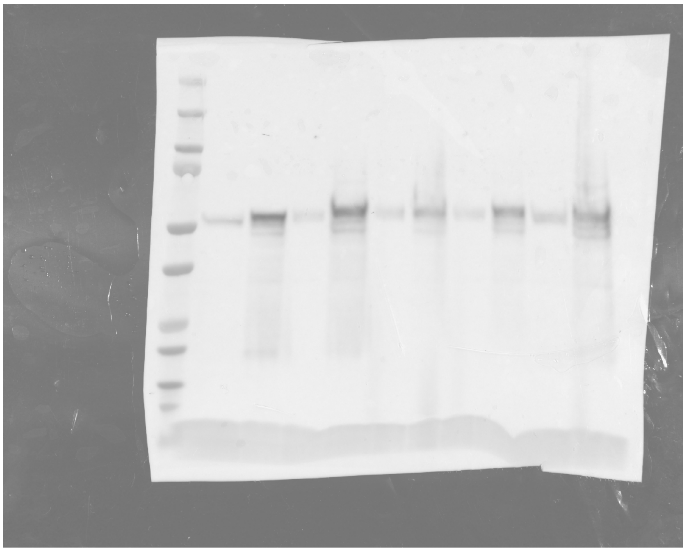

Actin

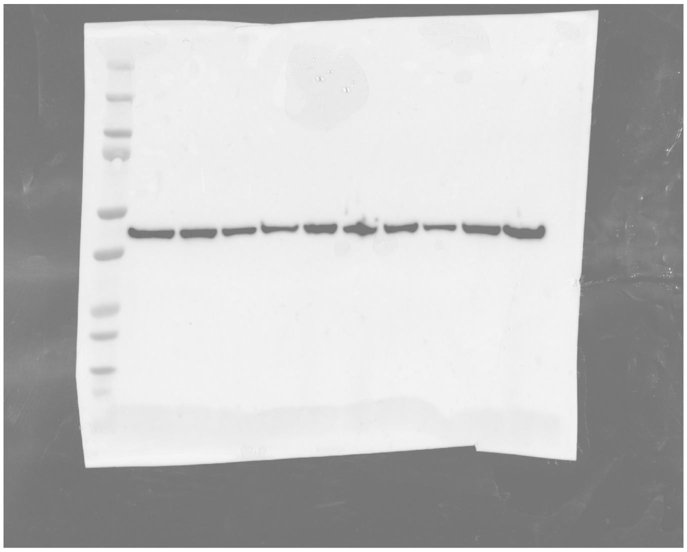

Figure 2d

p65

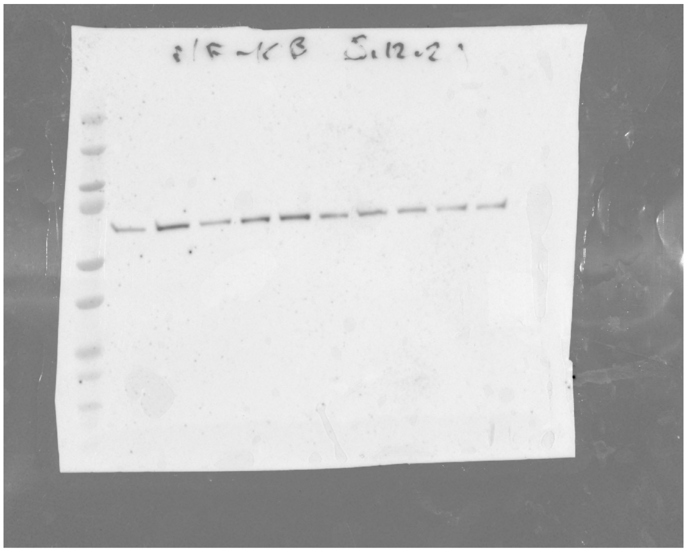

p-IkB

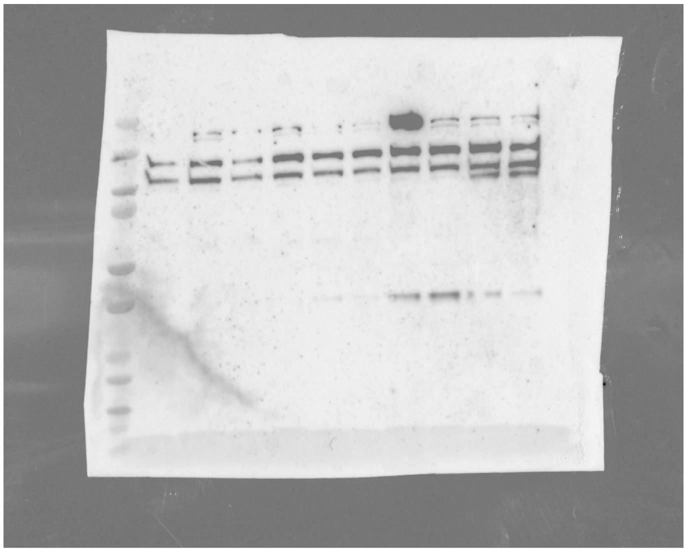

IkB

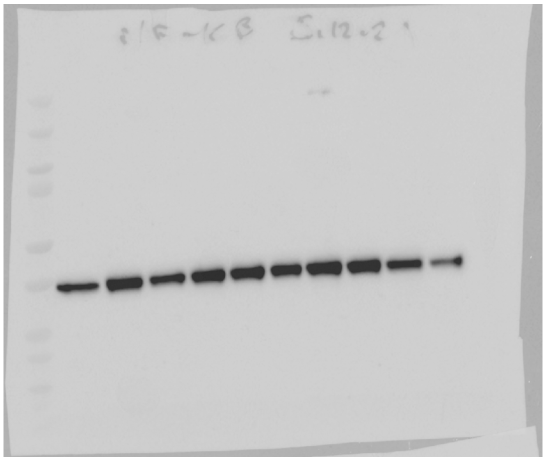

RIPK3

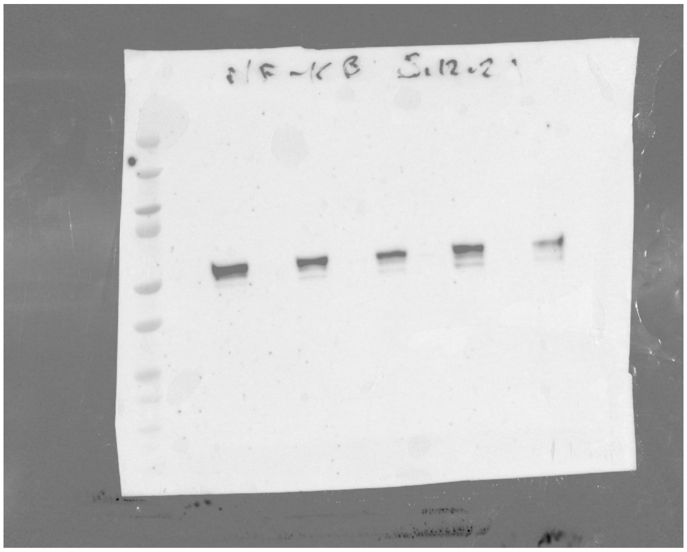

Actin

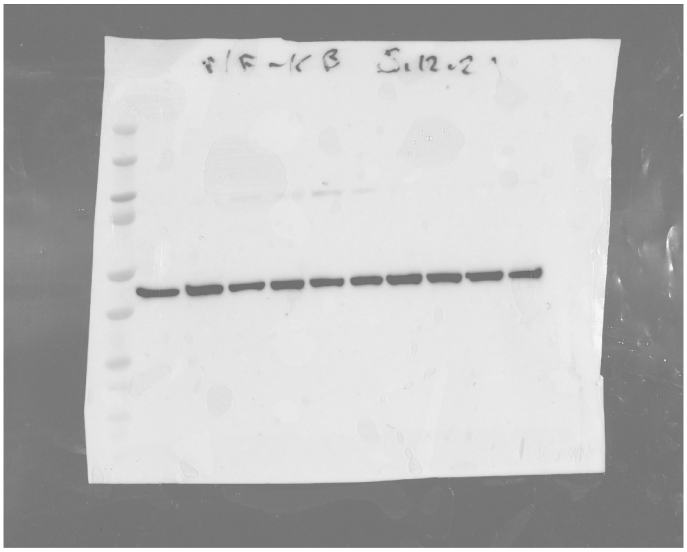

Figure 2f

pMLKL

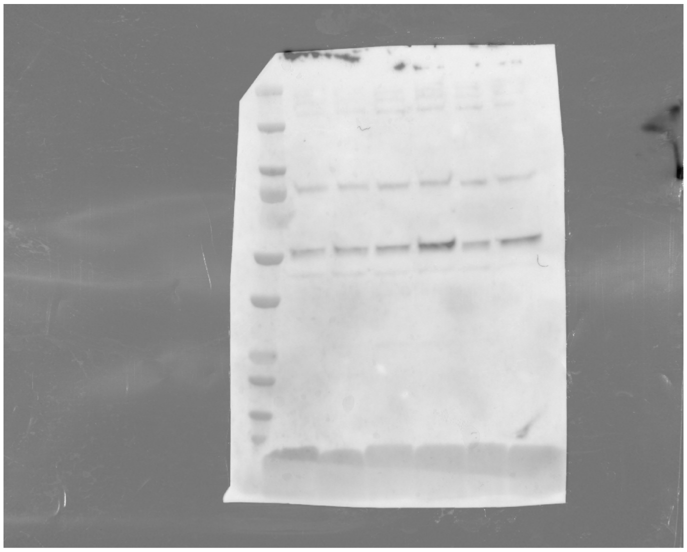

MLKL

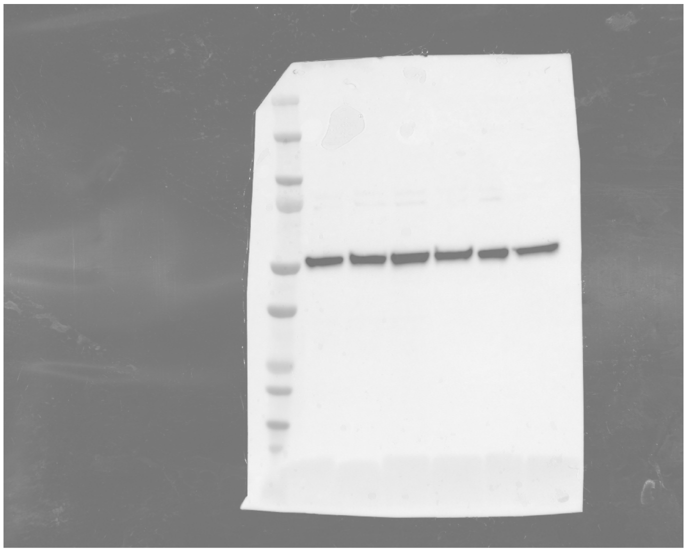

RIPK3

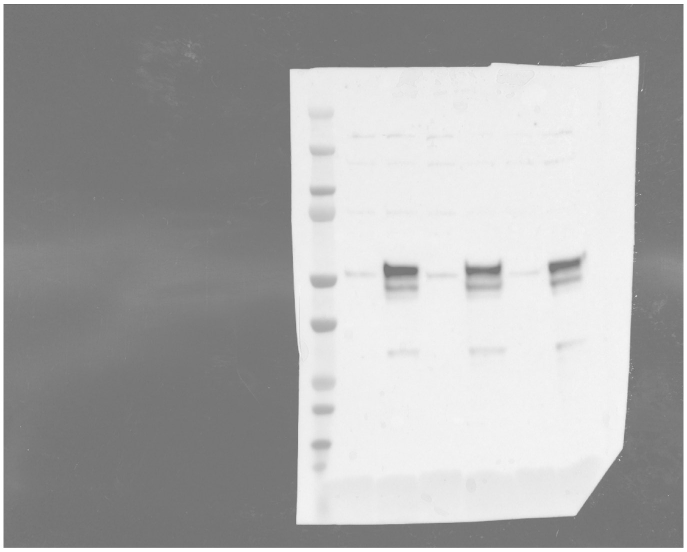

RIPK1

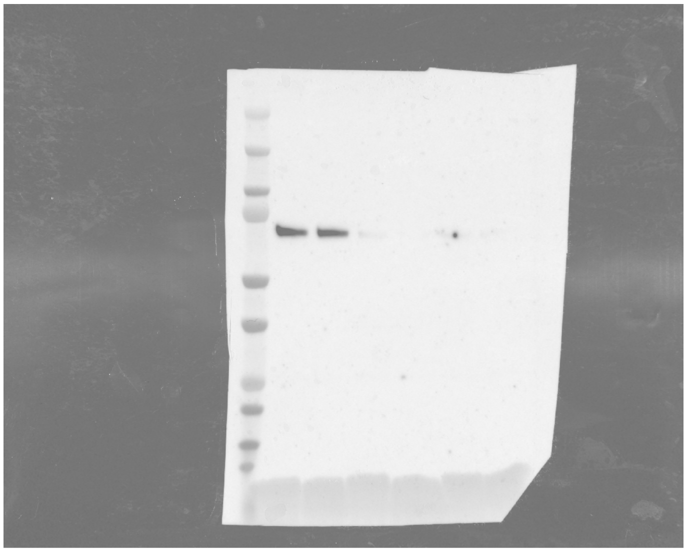

Actin

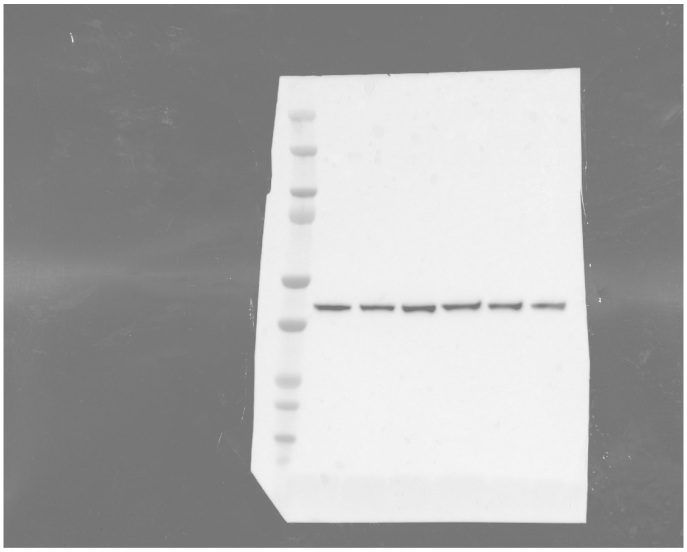

Supplement: Supplementary file 2 — Uncropped Western blot images [file 41419_2024_6801_MOESM2_ESM.pdf]
